# Editorial Comment from Dr Kobayashi to A case of hypogonadotropic hypogonadism due to hypophysitisdiscovered by secondary male infertility

**DOI:** 10.1002/iju5.12558

**Published:** 2022-11-08

**Authors:** Hideyuki Kobayashi

**Affiliations:** ^1^ Department of Urology Toho University School of Medicine Tokyo Japan

The risk of secondary male infertility increases with aging and adult‐onset hypopituitarism is very rare. In cases of hypogonadotropic hypogonadism, luteinizing hormone (LH), follicle‐stimulating hormone (FSH), and testosterone levels are minimal and azoospermia is present. A LH‐releasing hormone test and human chorionic gonadotropin (HCG) test are needed to diagnose hypogonadotropic hypogonadism. It is treated by hormone replacement therapy with injected HCG and recombinant FSH.

The authors report a case of hypogonadotropic hypogonadism due to lymphocytic hypophysitis diagnosed in a patient with secondary male infertility as the main complaint.^1^

They state that the cause of the secondary azoospermia in this case was hypophysitis.^1^ Secondary azoospermia may be associated with various events. For example, in their study on secondary azoospermia occurring after sleeve gastrectomy, Gomez *et al*. concluded that the secondary azoospermia was related to rapid weight loss.^2^ However, the present case report did not observe rapid weight loss.

The mechanism of hypophysitis is unknown and an association with the autoimmune mechanism is suspected. It would be of interest to elucidate the mechanism of hypophysitis.

This is the first report of hypogonadotropin hypogonadism due to hypophysitis discovered in secondary male infertility.^1^ When we examine patients with secondary male infertility in the clinical setting, we have to keep hypogonadotropic hypogonadism in mind, although it is rare.

Finally, the authors did not confirm recovery of spermatogenesis after the hormone therapy. They should have paid more attention to spermatogenesis in this case.

## Conflict of interest

The authors declare no conflict of interest.

## Author contribution

Hideyuki Kobayashi: Writing – original draft.
